# Complex somatic mutation landscape in myeloid cells in a patient with VEXAS syndrome: First Brazilian case report

**DOI:** 10.1016/j.htct.2024.05.013

**Published:** 2024-09-07

**Authors:** Fabíola Reis de Oliveira, Adriane Souza Lima, Carlos Roberto Faria, Thaise Oliveira Quaresma, Marcio M. Mourani, Lauro Wichert-Ana, Paulo Louzada, Fernanda Gutierrez-Rodrigues, Neal S. Young, Rodrigo T. Calado

**Affiliations:** a Department of Internal Medicine Ribeirão Preto Medical School, University of São Paulo, Ribeirão Preto, Brazil; b Department of Medical Imaging, Hematology and Oncology, Ribeirão Preto Medical School, University of São Paulo, Ribeirão Preto, Brazil; c Hematology Branch, National Heart, Lung, and Blood Institute, National Institutes of Health, Bethesda, MD, USA

VEXAS (**V**acuoles, **E**1 enzyme, **X**-linked, **A**utoinflammatory, **S**omatic) syndrome is a recently described autoinflammatory and hematologic disorder caused by somatic mutations in the *UBA1* gene (located in the X chromosome), which encodes the ubiquitin-activating enzyme 1 required for ubiquitination process pathways. In VEXAS, somatic mutations are acquired in hematopoietic stem cells but ultimately restricted to myeloid cells in peripheral blood; recurrent mutations are mostly seen in the p.M41 hotspot, although other variants have also been reported.^1^^,^^2^

Clinically, this disease is seen in older men with only seven female cases reported.^3^^,^^4^ It can present as a spectrum of different adult-onset autoinflammatory manifestations such as relapsing polychondritis, Sweet syndrome, giant cell arteritis and vasculitis. Patients also present hematologic findings: macrocytic anemia and thrombocytopenia are seen in most patients with up to 50 % being diagnosed with myelodysplastic neoplasms (MDS) and plasma cell dyscrasias, although its neoplastic nature is not fully understood. Furthermore, myeloid and erythroid precursors in the marrow display intracellular cytoplasmic vacuoles and the bone marrow is frequently hypercellular.^5^, ^6^, ^7^ Vascular thrombotic events are prevalent, in about 50 % of cases, and pulmonary disease such as alveolar hemorrhage and pleural effusions are commonly described. The description of many additional cases after the first reported case suggests that VEXAS is more prevalent than initially anticipated.

Here we report a single case of VEXAS in a Brazilian patient with an atypical molecular profile diagnosed after two years of chronic inflammatory disease refractory to multiple therapies. A 65-year-old man was initially evaluated at the rheumatologic clinic of a university hospital with an eight-month history of cyclic fever, relapsing auricular swelling and erythema, persistent skin lesions, and arthralgia. An ear biopsy confirmed auricular chondritis. The patient had macrocytic anemia (hemoglobin: 8.3 g/dL; mean corpuscular volume: 106 fL), mild thrombocytopenia (104 × 10^9^/L), and leukopenia (2.73 × 10^9^/L). The skin biopsy revealed dense inflammatory infiltration composed of neutrophils and lymphocytes, and vasculitis. An additional laboratory assessment showed an elevated erythrocyte sedimentation rate (ESR: 134 mm/h; upper limit [UL]; 20 mm/h) and C-reactive protein (17.1 mg/dL; UL: 1 mg/dL). Immunoassays for autoimmune and infectious diseases were inconspicuous, including negative for rheumatoid factor, and non-reactive for anti-citrullinated protein, antiphospholipid, anti-neutrophil cytoplasmic and antinuclear antibodies.

After the diagnosis of relapsing polychondritis, the patient was treated with oral prednisone (60 mg/day) and azathioprine 100 mg/day. Glucocorticoid tapering, however, repeatedly led to flares and new symptoms, such as weight loss, weakness, and scleritis. One year after the initial therapy, he developed sudden abdominal pain, and superior mesenteric vein thrombosis with celiac trunk dissection were found in the computed tomography angiography, with no evidence of microaneurysms, stenosis, large vessel vasculitis, or solid tumors.

A skeletal survey using ^18^fluorodeoxyglucose positron emission tomography (FDG-PET CT) demonstrated diffuse bone marrow hypermetabolism. Bone marrow aspirate showed significant vacuolated myeloid precursors, erythroid dysplasia but conventional cytogenetics showed normal karyotype (46, XY [20]). The diagnosis of a single-lineage myelodysplastic neoplasm was established.

Ferrada et al.^8^ had previously suggested the investigation of *UBA1* somatic mutations in male patients with relapsing polychondritis, high mean corpuscular volume and low platelet count. Based on the autoinflammatory, dermatologic, and hematologic findings, VEXAS syndrome was suspected as the cause of the patient's illness. Molecular diagnosis of VEXAS relies on the detection of loss-of-function *UBA1* mutations, which is not widely available in Brazil due to the cost and limited availability of next generation sequencing (NGS).

A collaborative effort allowed us to screen this patient for somatic mutations in *UBA1* and other myeloid cancer genes, which confirmed the VEXAS diagnosis. In peripheral blood, we identified the *UBA1* p.Met41Leu (c.121A>C) mutation at a variant allele frequency (VAF) of 0.51, two mutations in *TET2* (c.3955-1G>A; VAF, 0.33 and c.3443A>G; VAF, 0.06), *DNMT3A* (c.8900G>A; VAF, 0.33), and *SF3B1* (c.2098A>G; VAF, 0.04). This combination of *UBA1* mutation with three other significant somatic mutations in myeloid cells has not been described before (Figure 1).

**Figure 1:**
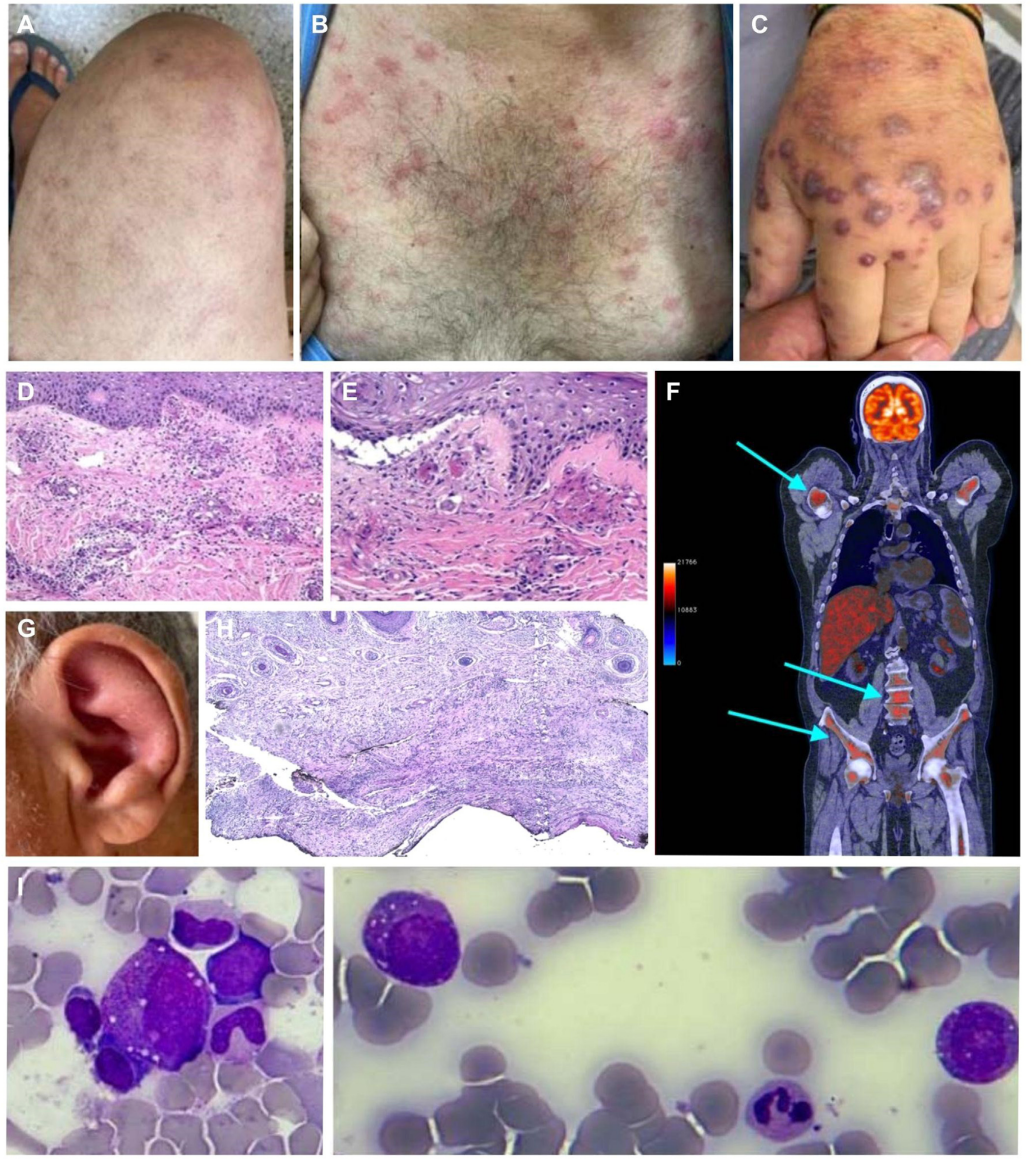
Clinical presentation of dermatological lesions: (A) livedo, (B) erythematous papules on the trunk and upper arms, (C) erythematous-purpuric infiltrated papules and nodules composing Sweet syndrome-like dermatosis. (D) & (E) Hematoxylin and eosin-stained sections of the skin biopsy specimen show a diffuse infiltrate of lymphocytes and neutrophils with leukocytoclasis in absence of eosinophils. There is evidence of vasculitis in superficial and deep vessels, fibrin deposits in the vascular wall, nuclear debris, and erythrocyte leakage. Bacterial, mycobacterial, and fungal cultures were negative. (F) 18F-fluorodeoxyglucose (FDG) positron emission tomography (PET-CT). (G) Auricular chondritis. (H) Hematoxylin and eosin-stained auricular biopsy specimens show lymph histiocytic infiltrate predominantly in the deep dermis with absence of vasculitis. (I) Bone marrow aspirates with myelodysplastic syndrome and vacuolated myeloid progenitor.

There is no consensus yet on the treatment of this disease.^9^ The patient failed to respond to corticosteroid-sparing therapies such as azathioprine, methotrexate and colchicine and remained dependent on intermediate doses of prednisone. In this scenario, therapy with antibodies against the interleukin-6 receptor was initiated. Clinically, there was an important improvement in the recurrence of skin lesions, including the frequency of auricular chondritis episodes. The most relevant laboratory changes after therapy are shown in Table 1.

**Table 1:** Comparison of hematological counts and inflammatory tests before treatment with monoclonal antibodies against interleukin-6 receptor (D 0), 20 days after the first dose (*D* + 20), and six months after monthly treatment with tocilizumab (*D* + 180).

	**Hemoglobin (g/dL)**	**MCV (fL)**	WBC (10^9^/L)	**Platelets (10^9^/L)**	**CRP (mg/dL)**	**ESR (mm/h)**
D 0	9.8	109	4.1	114	5.23	>140
*D* + 20	9.0	110	3.3	178	<0.40	24
*D* + 180	9.4	115	2.7	159	<0.40	16

MCV: mean corpuscular volume; WBC: white blood cells; CRP: C-reactive protein; ESR: erythrocyte sedimentation rate.

In a cohort of 83 cases^8^ with *UBA1* mutations, 26 (31 %) were diagnosed with MDS with excess blasts being found in only one case; three had abnormal karyotypes; somatic mutations were more common in the *DNMT3A* gene but in isolation. Only one case had concomitant *TET2 and DNMT3A* mutations. Our patient presented a peculiar combination of epigenetic (*TET2* and *DNMT3A*) and splicing (*SF3B1*) gene mutations. Noteworthy, recent data indicate that *TET2*-mutant clones are more prone to clonal expansion over time in comparison to other somatic mutations in a process driven by inflammation, especially via IL-1 signaling.^10^

The association between VEXAS and MDS is still not well understood.^11^^,^^12^ In this specific case, the *SF3B1* mutation may contribute to erythroid dysplasia in the bone marrow. Beck et al. showed increased IL-1β in an *UBA1b* deletion zebrafish model.^1^ Hypothetically, the autoinflammatory state in VEXAS may contribute to the appearance and expansion of *TET2*-mutated clones. The series of myeloid neoplasia-associated somatic mutations in this case reinforces the neoplastic characteristics of marrow involvement.

Further clinical screening for VEXAS showed no evidence for monoclonal gammopathy either by serum protein electrophoresis or by marrow flow cytometry.

Here, we report a single case of VEXAS in a man with an atypical molecular profile and a very typical disease history. Most reports describe the disease in patients from North America, Europe, and Japan with virtually no information from developing countries, which have distinct ethnic and genetic backgrounds.^2^ Even though the tool for confirming the diagnosis (NGS) is still almost unavailable in the Brazilian reality, awareness of the existence of this condition by hematologists, dermatologists and rheumatologists is essential to reduce underdiagnosis and consequently lead to a better understanding of the pathophysiology.

## Conflicts of interest

The authors declare no conflicts of interest
